# Identifying single-nucleotide polymorphisms responsible for the linkage signal of rheumatoid arthritis on chromosome 6 by joint modeling of linkage and association

**DOI:** 10.1186/1753-6561-1-s1-s40

**Published:** 2007-12-18

**Authors:** Wan-Yu Lin, Daniel J Schaid

**Affiliations:** 1 Institute of Epidemiology, National Taiwan University, Floor 5, No. 17, Shiujou Road, Zhongzheng District, Taipei 100, Taiwan; 2 Division of Biostatistics, Mayo Clinic, 200 First Street SW, Rochester, Minnesota 55905, USA

## Abstract

This study evaluated the utility of unrelated controls and flanking markers when performing joint modeling of linkage and association by the LAMP software (version 0.0.6) [*Am J Hum Genet *2005, 76:934–949; *Am J Hum Genet *2006, 78:778–792]. Analyses were conducted on the simulated rheumatoid arthritis (RA) data in Genetic Analysis Workshop 15 (GAW15), using single-nucleotide polymorphisms (SNPs) on chromosome 6 over the 100 simulated replicates. We found that the LOD score for testing association in the presence of linkage dramatically increased when unrelated controls were added to affected sib pairs (ASPs), and that choosing a sufficient number of flanking markers is critical in order to distinguish between perfect linkage disequilibrium (which leads to the conclusion of a measured SNP explaining a linkage signal) and incomplete linkage disequilibrium (which leads to the conclusion of other undetected causal variants in a linkage region).

## Background

The DRB1 alleles located in the HLA region of chromosome 6 have been found to affect susceptibility to rheumatoid arthritis (RA), and linkage at HLA was confirmed by several studies [1-4]. However, because linkage measures the effect of a large chromosomal region, follow-up association studies are needed to identify a causative locus. Traditional association studies do not distinguish a causal gene and a gene with indirect effects through linkage disequilibrium (LD); rather, they test the presence of LD, instead of the presence of perfect LD. Among the measures of LD that have been proposed for two-locus haplotype data, the two most common are |*D'*| and *r*^2 ^[5,6]: |*D'*| = 1 when the deviation of a haplotype frequency from randomly associated alleles attains its maximum value, given the marginal allele frequencies; *r*^2 ^= 1 when two single-nucleotide polymorphisms (SNPs) are perfectly correlated, sometimes called "perfect LD". This can arise when two SNPs arose on the same branch of the genealogy and remain undisrupted by recombination. In contrast, *r*^2 ^can have a value less than 1 when SNPs arose on different branches, or if an initially strong correlation has been disrupted by crossing over [7]. Here we distinguish between perfect LD (*r*^2 ^= 1) and complete LD (|*D'*| = 1).

To identify genes perfectly associated with disease, Li et al. [8] proposed a method to jointly model linkage and association, such that it can detect a marker's ability to explain a linkage signal, either partially or fully. They also discussed efficient study designs to test for association using sibship and unrelated individuals [9]. They found that when the disease is influenced by multiple genes, affected sib pairs (ASPs) provide more association information than singleton cases. Furthermore, a case-control study design can help to detect genes with small effects in the presence of genes with much larger effects.

To study the power of association tests in a linked region with different study designs, we used the SNP data on chromosome 6 for all 100 simulated replicates, and evaluated designs that either use only ASPs or combine ASPs with controls. All analyses were conducted with the software LAMP (version 0.0.6). We studied the simulated RA data with answers, to compare the LOD scores provided by LAMP under the two designs.

## Methods

### Phenotype, genotypes, and map

Using RA affection status as a binary trait, there were 1500 families with one ASP and their parents, all genotyped on 674 SNPs along chromosome 6. Additionally, 2000 unrelated controls were included in each replicate. There were no missing data or genotype errors. For the LAMP analyses, we specified the lifetime prevalence of RA to be 0.0107 as stated in the introduction of the simulated RA data. The sex-averaged map locations (in Haldane centimorgans) were used for the maps.

### Four analysis models

The software LAMP was used to fit four models by maximum likelihood: 1) a base model (BM) for no linkage and no association, *L*(*θ *= $\frac{1}{2}$, *r*^2 ^= 0), where *θ *is the recombination fraction and *r*^2 ^is the measure of LD. LAMP estimates marker allele frequencies with the assumption of Hardy-Weinberg equilibrium, and there is only one fitted parameter for each SNP; 2) a linkage equilibrium model (LE) for linkage without association, *L*(*θ *= 0, *r*^2 ^= 0). LAMP estimates disease and marker allele frequencies, as well as the penetrances for disease genotypes. The estimation of these parameters is constrained by the assumed disease prevalence; 3) a general model (GM) for linkage with any level of association, *L*(*θ *= 0, 0 <*r*^2 ^< 1). This most general model estimates three marker-disease haplotype frequencies and the penetrances for disease genotypes; 4) a linkage disequilibrium model (LD) for linkage with complete association, *L*(*θ *= 0, *r*^2 ^= 1). For this model, one of the marker alleles is assumed to directly affect disease susceptibility. The marker allele frequency and the penetrances for disease genotypes are estimated. The four models are summarized in Table 1.

**Table 1:** Four models compared in the three likelihood-ratio tests

Model^a^		Likelihood	d.f.	Parameters^b^	LR tests
(1)	BM	*L*(*θ *= $\frac{1}{2}$, *r*^2 ^= 0)	1	*p*_*A*_	
(2)	LE	*L*(*θ *= 0, *r*^2^= 0)	4	*p*_*A*_, *p*_*D*_, *f*_*DD*_, *f*_*Dd*_, *f*_*dd*_	(2) vs. (1): test for linkage
(3)	GM	*L*(*θ *= 0, 0 <*r*^2 ^< 1)	5	*p*_*DA*_, *p*_*Da*_, *P*_*dA*_, *f*_*DD*_, *f*_*Dd*_, *f*_*dd*_	(3) vs. (2): test for association in the presence of linkage
(4)	LD	*L*(*θ *= 0, *r*^2 ^= 1)	3	*p*_*A*_, *f*_*DD*_, *f*_*Dd*_, *f*_*dd*_	(3) vs. (4): test for other linked variants

^a^BM, base model; LE, linkage equilibrium; GM, general model; LD, linkage disequilibrium.

^b^*P*_*A *_= marker allele frequency; *P*_*D *_= disease allele frequency; *f*_*g *_= *P*(*affected | g*), *g *∈ {*DD*, *Dd*, *dd*}; *p*_*DA*_, *p*_*Da*_, *p*_*dA *_are the marker-disease haplotype frequencies.

### Linkage and association tests

The four models were used to create three likelihood ratio tests, and hence LOD scores: 1) a test for linkage, 2) a test for association in the presence of linkage, and 3) a test for other linked variants. In contrast to traditional parametric linkage analyses that specify a genetic disease model, LAMP calculates parametric maximized LOD scores by maximizing over all assumed model parameters. The method allows LD between the measured candidate SNP and an unobserved disease allele, but assumes linkage equilibrium between the flanking markers and the measured candidate SNP [8]. Note that this assumption may not hold for high-density SNP data. For the 674 SNPs on chromosome 6, there were 62 marker clusters that had large within-block LD, identified by setting the *r*^2 ^threshold to 0.4 in Merlin (version 1.0.1, with a command "*merlin -d datfile -p pedfile -m mapfile --npl --grid 1 --rsq 0.4 --cfreq*") [10,11]. To avoid problems of LD between the candidate SNP and the flanking markers, we eliminated SNPs that had large LD values by selecting a tag-SNP from each cluster. In each marker cluster, we kept the SNP with the highest marker heterozygosity (calculated by pedstats, version 0.6.3), and deleted other SNPs in the same cluster. There were 581 tag-SNPs after removing those with large LD.

### Two study designs

We considered two study designs: 1500 ASPs alone (ASPs), and 1500 ASPs with unrelated 2000 controls (ASPs+controls). The combined data should be more powerful to detect association, but not linkage. Our aims were to evaluate the increase in LOD scores provided by controls for the test for association (in the presence of linkage), and to evaluate the benefit of flanking markers for the test for other linked variants.

## Results

### Contribution of unrelated controls to LOD scores

**Figure 1:**
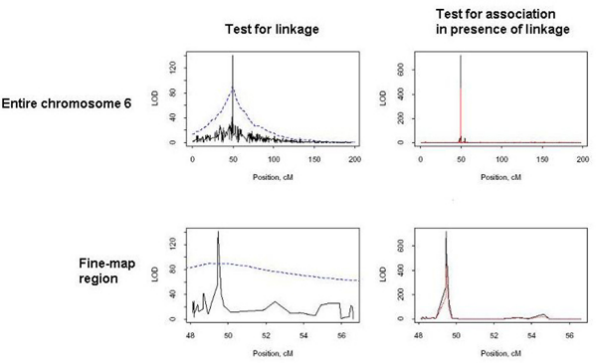
**LOD scores for test of linkage and test of association in the presence of linkage**. Simulated Replicate 45, representative of most 100 replicates, was randomly selected to illustrate key features. For the tests of linkage (left column), the black solid lines are the LOD scores obtained without the flanking markers, while the blue broken lines are the LOD scores obtained with a grid of flanking markers in the surrounding 30-cM region. For the tests for association in the presence of linkage (right column), the black solid lines are the LOD scores for ASPs+controls, while the red broken lines are the LOD scores for ASPs.

Results from the simulated replicates were very consistent, as summarized in Table 2, including the mean and range of LOD scores at SNP6_153 and SNP6_162 across the 100 replicates. These two SNPs were selected because they caused peaks for the test of association in the presence of linkage (see the second column of Figure 1). The peak linkage was at SNP6_153 (49.461 cM) in 75 out of 100 replicates, and at SNP6_154 (49.466 cM) in the other 25 replicates. Note that the two study designs resulted in the same LOD scores for linkage, since unrelated controls provided no information for linkage. The LOD score for association in the presence of linkage showed an extremely large value at SNP6_153 in all replicates for both study designs. Another signal was detected at SNP6_162 (54.625 cM). From Table 2, we can see that controls increased the LOD scores for the tests for association in the presence of linkage by approximately 60%. Furthermore, for SNP6_153, adding controls significantly increased the LOD score for the test for other linked variants, suggesting that SNP6_153 is not in perfect LD with a disease allele, and hence, that there may be other associated alleles in the region. This result agrees with the simulation scenario. In the simulated data, none of the 674 SNPs on chromosome 6 was in perfect LD (*r*^2 ^= 1) with the causal locus.

**Table 2:** Mean LOD scores for the three tests summarized from 100 replicates

	Test for linkage	Test for association in the presence of linkage	Test for other linked variants	
Models compared	LE vs. BM	GM vs. LE	GM vs. LD	

d.f.	4 - 1 = 3	5 - 4 = 1	5 - 3 = 2	

SNP6_153			With flanking markers	Without flanking markers
			
ASPs+controls		705.3 (range: 653.1–763.0)	7.8 (range: 2.2–17.9) (power^a^: 100%)	6.8 (range: 1.8–15.5) (power: 100%)
ASPs	132.3 (range: 105.9–152.9)	441.9 (range: 401.3–485.8)	4.1 (range: 0.8–12.6) (power: 94%)	1.7 (range: 0.0–5.3) (power: 53%)
SNP6_162				
ASPs+controls		44.4 (range: 30.0–62.2)	59.1 (range: 39.5–81.1) (power: 100%)	2.0 (range: 0.0–6.3) (power: 63%)
ASPs	9.1 (range: 3.4–15.8)	27.8 (range: 14.8–41.3)	58.9 (range: 39.7–81.0) (power: 100%)	1.8 (range: 0.0–6.1) (power: 55%)

^a^Power based on significance level of 0.05 and asymptotic chi-square distribution.

### Contribution of flanking markers to LOD scores

In addition to the impact of unrelated controls, flanking markers can have a large impact on LOD scores. Flanking markers smooth the LOD scores of the test for linkage, as we can see from the first column of Figure 1. Moreover, they are necessary to distinguish between perfect LD versus other linked variants, because this test relies on the evidence for linkage in the chromosomal region. To evaluate the impact of flanking markers, we used the SNPs located from 40 to 60 cM as the flanking markers, although only tag-SNPs were used. This region was highlighted because it presented a strong linkage signal, as we can see from Figure 1 (blue line in the upper-left plot). The black line in the first column of Figure 1 illustrates that the LOD scores for linkage in the linked region were drastically reduced when flanking markers were not used. Table 2 illustrates that the LOD scores for other linked variants were much too small when flanking markers were not used, giving the misleading conclusion that these two SNPs are in perfect LD with a disease causing allele.

## Discussion and conclusion

The joint modeling of linkage and association proposed by Li et al. [8] uses genotype information contributed by both the candidate SNP and the flanking markers, which they claimed outperforms the method of Göring and Terwilliger [12]. Their method identifies association in the presence of linkage, and can sometimes distinguish between perfect LD (leading to the conclusion of a causative SNP) and incomplete LD (leading to the conclusion of other causal variants in the region). A limitation is the assumption of linkage equilibrium between the candidate SNP and the flanking markers. This assumption is hard to meet with high-density SNP data. One way to eliminate LD among markers is to select tag-SNPs from each marker cluster. Further research to improve use of markers with LD would be worthwhile, recognizing that this might increase computational intensity.

In our analyses, we used sex-averaged maps without considering the sex differences in genetic map distances. The results of testing for linkage, testing for association in the presence of linkage, and testing for other linked variants were consistent with the simulation answers. However, ignoring sex differences in genetic maps may not lead to accurate inferences when genotypes for only one parent of each ASP are available [13]. A drawback of LAMP is that it is computationally demanding to search for the maximum likelihood estimates, particularly when pedigree data and microsatellite markers are used. To increase power to test for association in the presence of linkage without much computational intensity, combining ASPs with unrelated controls is a worthwhile design. Finally, our results emphasize the importance of choosing a sufficient number of flanking markers to distinguish between perfect LD and incomplete LD.

## Competing interests

The author(s) declare that they have no competing interests.
